# Development of Methods for the Mapping of Utilities Using Mixture Models: Mapping the AQLQ-S to the EQ-5D-5L and the HUI3 in Patients with Asthma

**DOI:** 10.1016/j.jval.2017.09.017

**Published:** 2018-06

**Authors:** Laura A. Gray, Mónica Hernández Alava, Allan J. Wailoo

**Affiliations:** School of Health and Related Research, University of Sheffield, Sheffield, UK

**Keywords:** EQ-5D, HUI, mapping, utility

## Abstract

**Background:**

Studies have shown that methods based on mixture models work well when mapping clinical to preference-based methods.

**Objectives:**

To develop these methods in different ways and to compare performance in a case study.

**Methods:**

Data from 856 patients with asthma allowed mapping between the Asthma Quality of Life Questionnaire and both the five-level EuroQol five-dimensional questionnaire (EQ-5D-5L) and the health utilities index mark 3 (HUI3). Adjusted limited dependent variable mixture models and beta-based mixture models were estimated. Optional inclusion of the gap between full health and the next value as well as a mass point at the next feasible value were explored.

**Results:**

In all cases, model specifications formally modeling the gap between full health and the next feasible value were an improvement on those that did not. Mapping to the HUI3 required more components in the mixture models than did mapping to the EQ-5D-5L because of its uneven distribution. The optimal beta-based mixture models mapping to the HUI3 included a probability mass at the utility value adjacent to full health. This is not the case when estimating the EQ-5D-5L, because of the low proportion of observations at this point.

**Conclusions:**

Beta-based mixture models marginally outperformed adjusted limited dependent variable mixture models with the same number of components in this data set. Nevertheless, they require a larger number of parameters and longer estimation time. Both mixture model types closely fit both EQ-5D-5L and HUI data. Standard mapping approaches typically lead to biased estimates of health gain. The mixture model approaches exhibit no such bias. Both can be used with confidence in applied cost-effectiveness studies. Future mapping studies in other disease areas should consider similar methods.

## Introduction

Preference-based measures (PBMs) that allow the calculation of health state utilities are not always administered in studies of clinical effectiveness. Nevertheless, these outcomes are often preferred by decision makers such as the National Institute of Health and Care Excellence to estimate quality-adjusted life-years (QALYs) for use in cost-effectiveness analysis [1]. “Mapping,” or “cross-walking,” is commonly used to estimate health state utilities when clinical studies have not included any PBM [2].

This article develops mapping methods and illustrates their use in relation to asthma. In clinical trials that include patients with asthma, the Sydney Asthma Quality of Life Questionnaire (AQLQ-S) is routinely recorded, but these trials often record no PBM and therefore QALYs cannot be estimated [3]. Nevertheless, there is increasing interest in how asthma is influencing health-related quality of life [4], [5]. For these reasons, studies have used mapping techniques to map from asthma-specific measures to PBMs [6], [7].

There are two broad approaches to mapping. The direct approach models the health state utility values themselves. The indirect approach, also referred to as response mapping, models each dimension of the PBM and calculates the predicted utilities as a second, separate step. Response-mapping models require observations (preferably a sizeable number) at all levels of each dimension and this can be a problem for small data sets if there are many different levels in each dimension.

Health state utility values are characterized by unusual distributions; they are commonly skewed, multimodal, and often have a large number of observations at 1 (indicating full health) and a gap between full health and the next feasible value. By definition, they are limited between the range of best and worst health states. Basic regression models are unable to capture all these features, which leads to biased estimates of health gain.

Beta regressions can provide flexibility when modeling skewed, bounded PBMs. Basu and Manca [8] proposed the use of single and two-part beta regressions to model PBMs and QALYs. The standard beta regression assumes that the dependent variable is defined only in the open interval (0,1) but many PBMs display negative values. Some studies have suggested that a beta regression is inappropriate in these cases [9]. Other studies have attempted to overcome this problem by converting ad hoc all negative values to 0 [7], [10], not only ignoring that some health states are worse than death but also potentially distorting the distribution because of the well-known sensitivity of beta regressions to pile-ups of values at the boundaries. Nevertheless, there is a standard transformation in the literature that allows the transformation of values in any open interval into a (0,1) interval [11]. After estimation, the expected value is then transformed to its original scale to obtain the correct predictions. In the area of mapping, this is the approach followed by Kent et al. [12] and Khan et al. [13]. Beta-based regression models have been found to be more robust and outperform linear regressions [8], [13], [14]. One significant issue when using beta regressions is how to deal with observations on the boundaries of the feasible utility range. Different methods have been proposed and it is recommended that the sensitivity of the estimates to the different methods be assessed [11]. Even though beta regressions can deal with the bounded nature of utility data and can reproduce various shapes, multimodality is difficult to capture.

Mixture models are increasingly being used in the context of mapping because of their flexibility and the ability to capture multimodality [7], [8], [9], [10], [11], [15], [16]. Mixtures of normal distributions have been used to model different PBMs such as the health utilities index mark 3 (HUI3) [17], the three-level EuroQol five-dimensional questionnaire (EQ-5D-3L) [12], [14], and the six-dimensional health state short form [14]. Some mixture models have been specifically designed for utility mapping such as the adjusted limited dependent variable mixture model (ALDVMM) [15], [16], [18], [19]. This uses a mixture of adjusted normal distributions to account for the multimodality of PBMs and includes a number of other useful characteristics. It contains built-in features that account for the peak of observations at full health and the option of a gap in the distribution below that peak. Other mixture models used for mapping include a mixture of Tobit models censored to account for the bounded nature of PBMs with an additional degenerate distribution at perfect health [20]. One additional study [13] claims to estimate a limited dependent variable model. Nevertheless, the model described is not a finite mixture model but a two-part model with an ad hoc assumption of a normal distribution for values of the dependent variable less than 0.3 and a beta binomial for values at 0.3 or higher. The split at 0.3 is justified via visual inspection of a kernel density plot of the dependent variable. Recently, beta mixture models have also been used in utility mapping with success [12]. In general, mixture models have been found to outperform nonmixture models [18], [19], [20]. One study found some evidence to suggest that beta regressions can outperform mixture models, which might be in part related to the distributional shape of the health utility measure being used [14].

This study develops knowledge about mapping methods by comparing approaches for estimating two PBMs, the five-level EQ-5D (EQ-5D-5L) and the HUI3, from the AQLQ-S score, a clinical asthma measure using data from an international sample [21]. Two different classes of mixture models are used: the ALDVMM and extensions to a beta mixture model [12], which 1) account for the gap in the PBM distributions between full health and the next feasible value and 2) allow alternative approaches to deal with observations on the boundary of the beta distribution [12]. We provide a choice of mapping algorithms for use in economic evaluation along with advice on how best to choose between them.

All models are estimated using user-written code in Stata (StataCorp, College Station, TX) via the commands “aldvmm” [18] and “betamix” [22].

## Methods

### Data

We used data from the Multi-Instrument Comparison (MIC) project data set, which includes data on 7933 observations across six countries: Australia, Canada, Germany, Norway, the United Kingdom, and the United States [21]. The data include information on well-being, health state utilities, and demographic characteristics. In addition, respondents who self-reported having specific conditions were asked to answer disease-specific questionnaires. In total, 856 respondents self-reported asthma and completed the AQLQ-S. Data were available for respondents’ age and sex as well as their EQ-5D-5L and HUI3 scores. After removing observations with missing values in any of the required variables, the final sample for analysis consisted of 852 observations.

### Preference-Based Measures

Both the EQ-5D-5L and the HUI3 are PBMs with health state utility estimates for each feasible response to their descriptive system. The EQ-5D-5L covers the same five dimensions as the original three-level version (mobility, self-care, usual activities, pain/discomfort, and anxiety/depression), but each dimension has five response levels (no problems, slight, moderate, severe, and extreme/unable to do). It is designed for self-completion, has a low response burden, and is applicable to a range of diseases and treatments. The HUI3 is also a self-completion questionnaire with eight dimensions (vision, hearing, speech, ambulation, dexterity, emotion, cognition, and pain). The levels for each dimension vary between 5 and 6. We use the value sets in the studies by Devlin et al. [23] and Furlong et al. [24] to attach utility values to each health state in the EQ-5D-5L and the HUI3, respectively. For both instruments, a value of 1 represents full health, a value of 0 is considered equivalent to being dead, and their values can be negative, representing a state worse than death. Both instruments have a gap between full health and the next feasible health state (these next feasible health states are 0.951 in the EQ-5D-5L and 0.97258 in the HUI3). We refer to this value as the truncation point; these are the highest possible values generated for each of the PBMs that are not represented by full health. The lower limits are −0.281 and −0.36, respectively, for the EQ-5D-5L and the HUI3.

### Asthma Quality of Life Questionnaire

The AQLQ-S was designed as a measure of quality of life for adult patients with asthma. The questionnaire contains 20 questions within four domains (symptoms, activity limitation, emotional function, and environmental stimuli). Each of the questions allows a response on a 0 to 4 scale, with 0 representing no problems at all. The scores for each question are averaged to produce an overall AQLQ-S score between 0 and 4. Although there are many different versions of the AQLQ, the AQLQ-S is recommended by the European Medicines Agency [25] and has been validated [26]. Nevertheless, because the scoring is not preference-based, it is not suitable for use in cost-utility analysis.

Comparison of the AQLQ-S with the EQ-5D-5L and the HUI3 is shown in Figure 2 of the study by Kaambwa et al. [7] The EQ-5D-5L has good overlap with the AQLQ-S. The only dimension of the EQ-5D-5L that is not covered directly by the AQLQ-S is pain/discomfort. The dimensions of the HUI3 have less overlap with the AQLQ-S. The social and concerns dimensions of the AQLQ-S are not represented by any dimensions of the HUI3. In addition, the vision, pain, hearing, speech, dexterity, and cognition dimensions of the HUI3 are not represented in the AQLQ-S. Nevertheless, correlations between both PBMs and the AQLQ-S are highly significant; they are −0.4837 for the EQ-5D-5L and −0.4572 for the HUI3, similar to those observed in previous studies [7].

### Statistical Methods

It was not feasible to conduct response mapping because this requires observations in each response category of the different dimensions covered by the target descriptive system. In the case of the EQ-5D, there were no observations with the worst possible response for self-care. For the HUI, there were no observations with the worst possible response for vision.

We compared two approaches to direct mapping, both based on mixture models. The first is an ALDVMM implemented using the publicly available Stata command “aldvmm” [18]. This model has previously been applied for mapping across a range of clinical areas, including rheumatoid arthritis [18], [19], osteoarthritis [27], ankylosing spondylitis [28], and traumatic brain injury [29]. It has been shown to outperform other methods (linear regression, Tobit, and response mapping). The ALDVMM is a bespoke model developed specifically for utility mapping and the Stata function includes a number of user-specified options to tailor the method to the target utility instrument and country-specific tariff of interest. This includes specifying the next feasible value after full health [18], the “truncation point,” thus creating the typical gap seen in PBMs. There is the option to specify no truncation and therefore allow each component of the mixture model to be fully continuous up to the highest feasible value of 1 for full health. The method has previously been described in detail [19]. In brief, ALDVMM is a mixture of adjusted, normal distributions for use when the dependent variable is limited above at 1 (full health) and below in this case at −0.205 for the EQ-5D-5L and −0.36 for the HUI3. Besides estimating the model with different numbers of components, we estimated it with and without truncation.

The second model we used was a beta-based mixture model estimated via the user-written Stata command “betamix” [22], which is a generalization of the truncated inflated beta regression model introduced in the study by Pereira et al. [30]. This is a two-part model consisting of a multinomial logit model and a beta mixture model. A beta distribution cannot deal with observations at the boundaries. The addition of the multinomial logit model to the beta mixture allows for these observations and a mass of observations at full health. The model assumes a limited dependent variable $y_{i}$ for each individual *i* defined at point 1 and the interval [a,τ], where a<τ<1 and can be written as follows:

(1) $$g\left(y_{i}|x_{i1},x_{i2},x_{i3}\right)=\{\begin{array}{c} P(y_{i}=a|x_{i3}),\;|y_{i}=a\\ \begin{array}{c} P\left(y_{i}=\tau |x_{i3}\right),\;y_{i}=\tau \\ \begin{array}{c} P\left(y_{i}=1|x_{i3}\right),\;y_{i}=1\\ \left[1-\sum_{s=a,b,\tau }P\left(y_{i}=s|x_{i3}\right)\right]h\left(y_{i}|x_{i1},x_{i2}\right),\;y_{i}\in \;(a,\tau ) \end{array} \end{array} \end{array}$$

with probabilities:

(2) $$P\left(y_{i}=k|x_{i3}\right)=\frac{\exp(x_{i3}^{\prime }\gamma_{k})}{1+\sum_{s=a,p,b}\exp(x_{i3}^{\prime }\gamma_{s})},$$

where $x_{i3}$ is a vector of variables influencing the probabilities, $\gamma_{k}$ is a vector of coefficients, and *s* refers to each section of the distribution. For identification, the coefficients corresponding to the continuous part of the distribution are set to 0. The probability density function for the continuous part of the distribution has probability density function h(⋅) made up of a mixture of C-components each representing a beta distribution, with mean $\mu_{c_{i}}$ and precision parameter $\phi_{c}$, where c=1,…,C, such that:

(3) $$h\left(y_{i}|x_{i1},x_{i2}\right)=\sum_{c=1}^{C}\left(P(c|x_{i2})f(y_{i}|x_{i1}\beta_{c},\phi_{c},a,\tau )\right),$$

where f(⋅) is a beta density with alternative parameterization and C is the number of components included in the analysis. Component membership is determined using a second multinomial logit model, such that:

(4) $$P\left(c|x_{i2}\right)=\frac{\exp(x_{i2}^{\prime }\delta_{c})}{\sum_{j=1}^{C}\exp(x_{i2}^{\prime }\delta_{j})},$$

where $x_{i2}$ is a vector of variables influencing the probability of component membership and $\delta_{c}$ is a vector of corresponding coefficients. Again, one set of coefficients is set to 0 for identification.

The model is not constrained to the (0,1) range but transforms values to the relevant interval for the target utility instrument (−0.281 to 1 for the EQ-5D-5L and −0.36 to 1 for the HUI3). It is capable of producing estimates at either of the feasible limits, although for health utilities this is most relevant for mass points at full health (1). As with the ALDVMM, betamix also allows the specification of a gap between full health and the next feasible health state and for a mass of observations at this truncation point. Although it is possible to include a probability mass at the lower utility limit, for both model types, we did not include this here because our sample contained no observations with values at the lower utility limit for either PBM.

We estimated different specifications of each model type, with different numbers of components and with and without probability masses at certain points of the distribution. We included the AQLQ-S summary score, age, age-squared, and sex as covariates in all parts of the model. We attempted to keep the number of independent variables to a minimum so as to keep the mapping algorithms more generalizable for use in a wider range of data sets. We also considered including the individual dimension scores of the AQLQ-S rather than the total score, but found that our results were not significantly improved.

For comparison, we also estimated EQ-5D-5L and HUI3 using linear regression. We used the same independent variables in these models to ensure the models were comparable. We also compared our results with those from Kaambwa et al. [7] who mapped the AQLQ-S onto both the EQ-5D-5L and the HUI3, among other health state utilities in patients with asthma using data from the MIC data set [7]. They used four simple methods: ordinary least squares, censored least absolute deviations, generalized linear model, and a beta binomial regression model.

Preferred models were selected using various fit statistics: Akaike and Bayesian information criteria (AIC and BIC), root mean squared error (RMSE), mean absolute error (MAE), and mean error (ME). We assessed the fit across the distribution of disease severity. We compared the conditional distribution function of the observed data with the one derived from the estimated model. This builds on previous work in the area, which focuses on the summary measures [7]. In many cases, each of these criteria supports different models and so judgment must be used in determining the preferred model.

## Results

The final sample consisted of 852 observations (see Table 1) of which 62.3% were from females. Age ranged from 18 to 89 years. Although the AQLQ spanned the entire range of feasible values (0–4), neither the EQ-5D-5L nor the HUI3 did.

**Table 1:** Sample summary statistics

	**Mean ± SD**	**Minimum**	**Maximum**
**AQLQ-S**	0.7085 ± 0.7766	0	4
**EQ-5D-5L**	0.8425 ± 0.1693	−0.073	1
**HUI3**	0.7560 ± 0.2408	−0.1958	1
**Age (y)**	43.03 ± 15.00	18	89
**Country**	**No. of observations (%)**		
**Australia**	141 (16.55)	–	–
**USA**	150 (17.61)	–	–
**UK**	150 (17.61)	–	–
**Canada**	138 (16.20)	–	–
**Norway**	126 (14.79)	–	–
**Germany**	147 (17.25)	–	–

AQLQ-S, Sydney Asthma Quality of Life Questionnaire; EQ-5D-5L, five-level EuroQol five-dimensional questionnaire; HUI3, health utilities index mark 3.

**Fig. 1:**
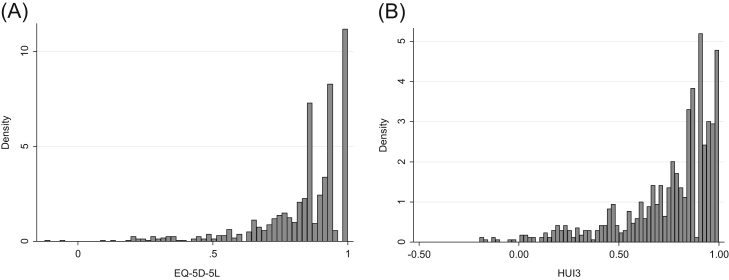
Distribution of (A) EQ-5D-5L and (B) HUI3. EQ-5D, EuroQol five-dimensional questionnaire; HUI3, health utilities index mark 3.

Figure 1A,B shows the distributions of the EQ-5D-5L and the HUI3, respectively. Both the HUI3 and the EQ-5D-5L exhibit mass points at the upper full-health limit: 20.9% in the EQ-5D-5L and 9.5% in the HUI3. For the EQ-5D-5L, there was no significant mass of observations at the truncation point (0.951). Almost 6% of observations were at the HUI3 truncation point (0.973). There were a relatively large number of observations with an EQ-5D-5L at 0.942, 0.924, and 0.866, associated with slight problems with anxiety and depression and/or pain and discomfort.

We found that models that formally included the gap between full health and the next feasible value outperformed those that did not, using both ALDVMM and beta-based mixture models. We therefore concentrated on comparing alternative specifications of models that included this gap. This was the case for estimating both the EQ-5D-5L and the HUI3.

Our preferred models are a three-component beta-based model with a probability mass at full health and a three-component ALDVMM when estimating the EQ-5D-5L. When estimating the HUI3, our preferred models are a four-component beta-based model with probability masses at full health and the truncation point and a four-component ALDVMM. Reasons for choosing these preferred models are discussed here.

All mixture models have the expected sign and produce simulated data that are good predictions of the sample data. The estimated coefficients for the four preferred models are displayed in the Appendix in Supplemental Materials found at doi:10.1016/j.jval.2017.09.017 along with a Stata .do file that allows users to enter their own data to predict EQ-5D-5L and HUI3 using these preferred models.

### Five-Level EuroQol Five-Dimensional Questionnaire

**Fig. 2:**
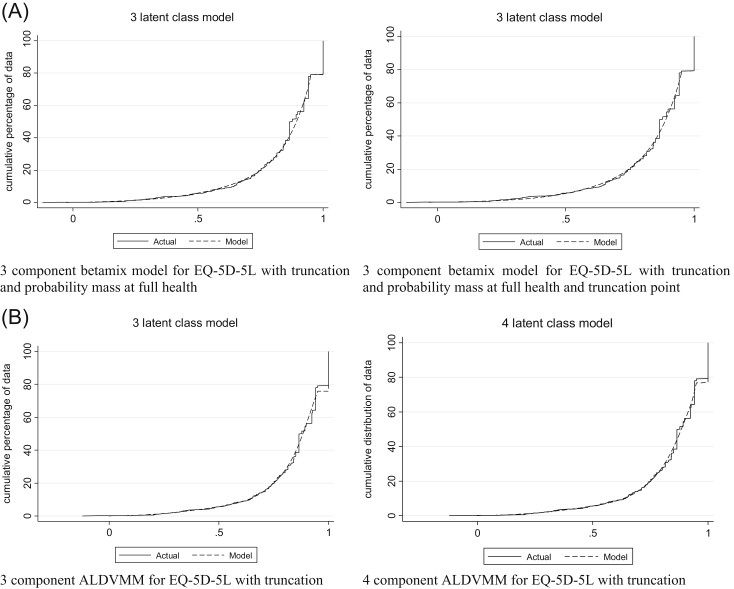
(A) Conditional distribution functions for models estimating EQ-5D-5L with betamix. (B) Conditional distribution functions for models estimating EQ-5D-5L with ALDVMMs. ALDVMM, adjusted limited dependent variable mixture models; EQ-5D-5L, five-level EuroQol five-dimensional questionnaire.

**Fig. 3:**
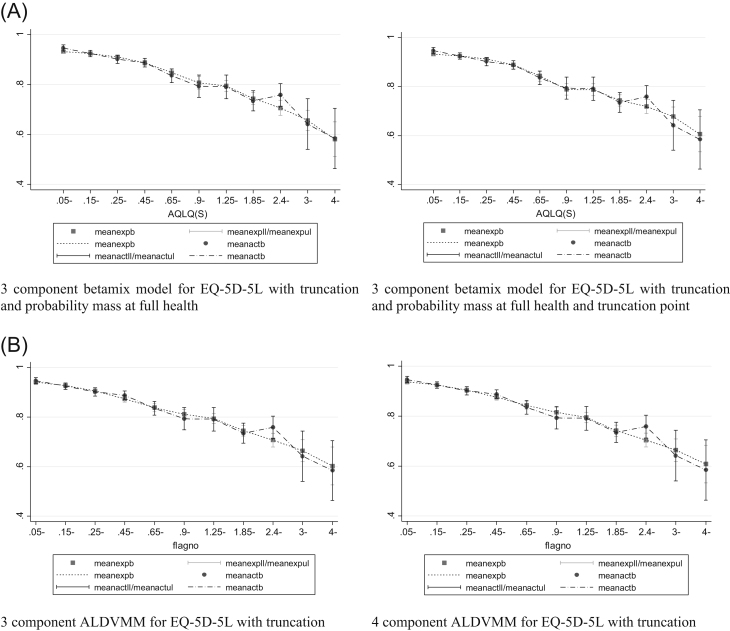
(A) Mean fit vs. mean observed PBM by the AQLQ for models estimating the EQ-5D-5L with betamix. (B) Mean fit vs. mean observed PBM by the AQLQ for models estimating the EQ-5D-5L with the ALDVMM. ALDVMM, adjusted limited dependent variable mixture models; AQLQ, Asthma Quality of Life Questionnaire; EQ-5D-5L, five-level EuroQol five-dimensional questionnaire; PBM, preference-based measure.

Beta mixture models required a specified probability mass at full health to ensure they estimated the correct proportion of observations at full health. Table 2 presents model performance criteria for three- and four-component models, each with inclusion of truncation, a mass point at full health, and with and without a further probability mass at the truncation point (0.951). Differences in measures of “error” between the three- and four-component models were small, with BIC lower for the three-component model [19]. The model that does not include a probability mass at the truncation point appears to better predict the lower end of the EQ-5D distribution. This can be seen in the conditional distribution function graphs in Figure 2A and the plots of mean predicted versus observed fit in Figure 3A. This is because there are a relatively small number of observations at the truncation point but a large proportion of observations at the value just below the truncation point (13.73% at 0.944). If this spike in observations was at the truncation point itself, the model that included a probability mass at the truncation point might have shown better fit. For these reasons, the optimal beta-based model has three components and a probability mass at full health but not at the truncation point.

**Table 2:** Model specifications and model choice criteria (n = 852)

**No. of components**	**Specification**	**Log likelihood**	**No. of parameters**	**RMSE**	**MAE**	**ME**	**AIC**	**BIC**
*EQ-5D-5L betamix*								
3	Probability mass at full health	527.68	33	0.1430	0.1001	−0.0003	−989.36	−832.69
3	Probability mass at full health and truncation point	436.00	38	0.1425	0.1003	0.0005	−796.00	−615.59
4	Probability mass at full health	538.87	44	0.1429	0.1002	0.0003	−989.75	−780.86
4*	Probability mass at full health and truncation point	401.81	45	0.1474	0.1018	−0.0012	−713.62	−499.98
*EQ-5D-5L ALDVMM*								
3	Bounded	322.42	28	0.1439	0.1006	0.0003	−588.84	−455.90
4	Bounded	336.64	39	0.1439	0.1004	0.00003	−595.28	−410.13
*HUI3 betamix*								
3	Probability mass at full health	708.05	33	0.2081	0.1566	0.0018	−1350.09	−1193.42
3	Probability mass at full health and truncation point	207.64	38	0.2081	0.1563	0.0024	−339.28	−158.88
4	Probability mass at full health	727.92	44	0.2076	0.1562	0.0017	−1367.80	−1158.95
4	Probability mass at full health and truncation point	224.48	49	0.2067	0.1548	0.0009	−350.96	−118.33
*HUI3 ALDVMM*								
3	Age included	192.51	28	0.2076	0.1556	0.00041	−329.03	−196.10
4	Age included	212.42	39	0.2071	0.1550	0.00033	−346.85	−161.69
3	No age in probability variables $x_{2}$	189.87	24	0.2082	0.1563	0.00054	−331.75	−217.80
4	No age in probability variables $x_{2}$	201.09	33	0.2082	0.1563	0.00026	−336.18	−179.51
*Linear model*								
-–	OLS-EQ-5D	424.21	5	0.1471	0.1023	−6.47 × 10^−17^	−838.42	−814.69
–	OLS-HUI3	112.35	5	0.2121	0.1598	3.23 × 10^−17^	−214.70	−190.96

*Note*. All models outlined here include a truncation at the best possible health state other than full health.

AIC, Akaike information criterion; ALDVMM, adjusted limited dependent variable mixture models; AQLQ, Asthma Quality of Life Questionnaire; BIC, Bayesian information criterion; EQ-5D-5L, five-level EuroQol five-dimensional questionnaire; HUI3, health utilities index mark 3; MAE, mean absolute error; ME, mean error; OLS, ordinary least squares; RMSE, root mean squared error.

⁎ This model would not converge with the AQLQ score in the probabilities parameters. The results presented here are for a model without AQLQ in the probabilities.

Results for the three- and four-component ALDVMMs are presented in Table 2. The four-component model offers improvements in RMSE, MAE, and ME but has a higher BIC. Figures 2B and 3B show that both models fit the data closely, suggesting that the three-component model is preferred. Figure 2B shows some disparity between the distribution from the model and the data at the upper end of the EQ-5D-5L. This occurs not at the full-health value (the data have 21% of observations here compared with 24% in the simulated data in the models) but at values just below the truncation point.

### Health Utilities Index Mark 3

**Fig. 4:**
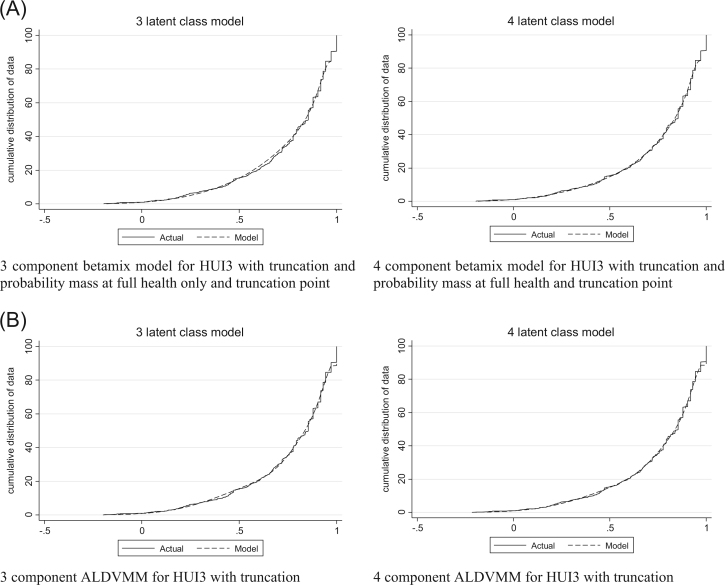
(A) Conditional distribution functions for models estimating the HUI3 with betamix (observed vs. simulated data [1000 observations]). (B) Conditional distribution functions for models estimating the HUI3 with the ALDVMM (observed vs. simulated data [1000 observations]). ALDVMM, adjusted limited dependent variable mixture models; HUI3, health utilities index mark 3.

**Fig. 5:**
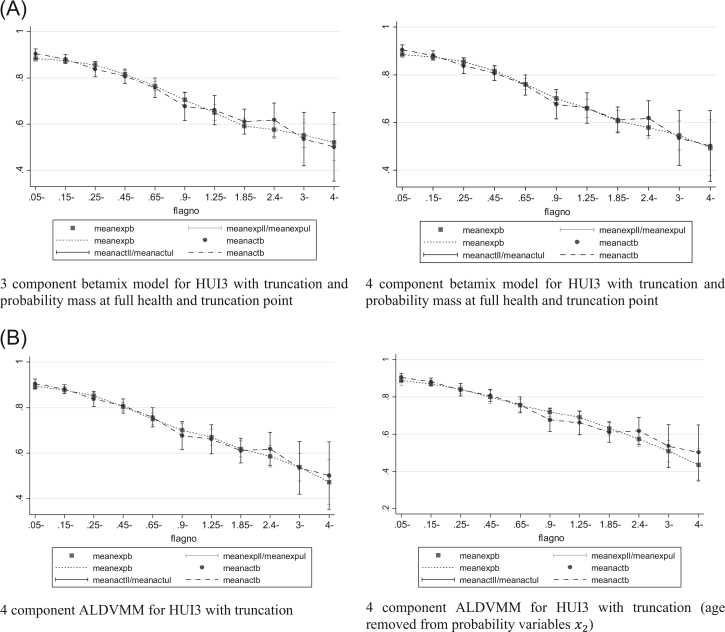
(A) Mean fit vs. mean observed PBM by the AQLQ for models estimating the HUI3 using betamix. (B) Mean fit vs. mean observed PBM by the AQLQ for models estimating the HUI3 using the ALDVMM. ALDVMM, adjusted limited dependent variable mixture models; AQLQ, Asthma Quality of Life Questionnaire; HUI3, health utilities index mark 3; PBM, preference-based measure.

Beta-based models without a probability mass at full health had difficulty fitting the correct number of observations at this value and so we report only those models that explicitly modeled this gap and include a probability mass at full health. The model that consistently produced the smallest errors was the four-component model with probability masses at full health and at the truncation point (0.973). Nevertheless, although the AIC suggested that the fourth component is beneficial, the BIC suggested that the three-component model is preferred. The simulated graphs in Figure 4A and the plots of the means in Figure 5A show a clear improvement in the model with the additional fourth component, particularly toward the lower end of the HUI3 distribution. We consider the four-component model to be the optimal beta mixture model.

As with the beta mixture models, the four-component ALDVMMs produced lower errors than did the three-component equivalents. In all the ALDVMMs estimating the HUI3, the coefficients for the variables influencing component membership were statistically insignificant, for example, age had *P* values all in excess of 0.03. We therefore investigated the use of different variables to predict component membership. Results are presented in Table 2 for models that do not include age to predict component probabilities as well as those that do. Although AIC and BIC generally favor the exclusion of age, other measures of error are worse. Figures 4B and 5B show a marked difference between these models and suggest that age should remain an explanatory variable for the probabilities because they considerably improve the fit of the model. The evidence indicates that the observed statistical insignificance associated with age may be related to the limited sample size.

### Comparison with Traditional Models

The performance criteria from the linear regression models are presented in Table 2. Although the linear regressions provide accurate estimates of the mean for both the EQ-5D-5L and the HUI3, they exhibit bias away from the center of the PBM distributions. This is shown by the RMSE and the MAE, which are larger than the preferred mixture models in both cases. Note that the AIC and the BIC cannot be compared between the linear regression models and the mixture models.

Table 3 presents the results from Kaambwa et al. [7] for models that include independent variables AQLQ-S, age, and sex. These specifications are chosen for comparison because they are most similar to the independent variables used in this study. Linear regression results found by Kaambwa et al. are very similar to those found in our study, suggesting that our results are comparable despite the small difference in sample size and the inclusion of the age-squared term in our study. In all cases, we find that our preferred models have lower RMSE than do each of the models used by Kaambwa et al.

**Table 3:** Results from Kaambwa et al. [7]

**Specification**	**RMSE**	**MAE**
*EQ-5D*		
OLS	0.1468	0.1027
CLAD	0.1491	0.1001
GLM	0.1463	0.1025
BB	0.1491	0.1051
*HUI3*		
OLS	0.2130	0.1608
CLAD	0.2188	0.1545
GLM	0.2120	0.1605
BB	0.2154	0.1643

BB, beta binomial; CLAD, censored least absolute deviations; EQ-5D, EuroQol five-dimensional questionnaire; GLM, generalized linear model; HUI3, health utilities index mark 3; MAE, mean absolute error; OLS, ordinary least squares; RMSE, root mean squared error.

## Discussion

We compared different mapping methods using data collected from people with asthma. Both the EQ-5D-5L and the HUI3 were considered as target utility instruments, from the AQLQ-S. We focused on direct mapping methods because response mapping was not feasible in this sample for either instrument. A move to the EQ-5D-5L, compared with the EQ-5D-3L, is likely to reduce the feasibility of applying response mapping in future, because data samples will more often fail to span all the levels described in the more detailed descriptive system.

Beta-based mixture models and ALDVMMs were estimated. Within these classes of model type we compared different numbers of components in the mixture models with and without a specified gap between full health and the next feasible value. For the beta-based models, where the gap was specified, we also considered whether there needed to be a specified probability mass at the truncation point.

Models with truncation to create a gap between full health and the next feasible value were universally preferred. This gap is part of the “bespoke” nature of both the ALDVMM and the beta-based mixture model, and although the importance of the method has been demonstrated in several studies using the EQ-5D-3L [19], the gap is more pronounced in that instrument (0.12). This contrasts with 0.049 and 0.027 in the EQ-5D-5L and the HUI, respectively. The next largest gap between feasible values is 0.024 in the EQ-5D-5L and 0.027 in the HUI3. To our knowledge, no mapping study that uses a mixture of beta distributions has accounted for the gap between full health and the next feasible utility value. We find that it remains important for mapping models to explicitly reflect this characteristic of utility instruments, even when the gap is relatively small. Others have claimed that the gap is sufficiently small not to warrant formal inclusion in the model [7]. Furthermore, the finding is relevant to all mapping methods and not simply those that use mixture model approaches.

Beta regression mixture models that better fitted both the EQ-5D-5L and the HUI3 than the ALDVMMs were identified. Preferred models for the HUI3 allowed an inflated number of observations at the next feasible value below full health. This feature adds further flexibility to the models but caution needs to be exercised in interpreting this finding. Theoretically, the addition of a mass point to the beta model is similar to adding an additional component to the ALDVMM. In this sense, the beta mixture approach is more artificial than the ALDVMM, and comparisons between models with the same number of components are not necessarily comparisons of like with like. The addition of mass points at selected points in the distribution does offer a means to improve fit, but this requires the addition of more parameters compared and is also less generalizable: there is a risk of overfitting to the data. In general, we would recommend caution in the inclusion of probability masses at *any* point in the distribution without some theoretical rather than empirical justification for doing so. The increased number of parameters required by the beta-based mixture model means that for smaller data sets there is a danger that it might be more difficult to identify. For example, we attempted to estimate the EQ-5D-5L using a four-component model with probability masses at full health and the truncation point, but this model would not converge when the AQLQ-S was included in the component membership probabilities. To reduce the number of parameters, we estimated a model without the AQLQ-S in the probabilities and achieved a much worse fit as a result.

Our results show the importance of considering the distribution when choosing the most appropriate model. The proportion of observations at the truncation point in a sample should be considered when choosing a model, in particular when using the beta-based mixture model. We found that when there were lots of observations at the truncation point (as with the HUI3), a probability mass needed including at the truncation point. The inclusion of this probability mass did not improve the model for the EQ-5D-5L, which had very few observations at the truncation point. We do not know whether this is generalizable to all applications of the HUI3 and the EQ-5D-5L or to other data sets. Further research is needed to determine the extent to which these results are generalizable.

We find that, in this case, both the beta-based mixture models and the ALDVMMs outperform linear regression. Linear regression can accurately predict the mean values of the PBM; it does not accurately predict values at the ends of the PBM distributions. We find that, using linear regression, PBMs are overestimated in individuals in poor health and underestimated in individuals in good health; this could have important consequences for cost-effectiveness estimates. This result is supported by a growing literature that suggests that mixture models outperform more traditional mapping techniques [18], [19], [20], [28], [31].

Other studies have been conducted using asthma outcomes. Tsuchiya et al. [6] estimated EQ-5D-3L from the less commonly reported 32-item AQLQ-McMaster score. In addition to direct mapping using a linear regression, this study carried out the first response mapping we are aware of. Our results are not directly comparable with this study because the AQLQ scores differ. We are able to compare our results with those of Kaambwa et al. [7] who used the same data as in our study. We compare our results with those that included the AQLQ-S total score, age, and sex. Our specification also included an age-squared term, and we had a slightly different sample size from that of Kaambwa et al. Nevertheless, the results using linear regression in our study are very similar to those from Kaambwa et al., reported in Table 3. This suggests that the results are reasonably comparable.

Kaambwa et al. found that of the four models they investigated, the generalized linear model was consistently their best-fitting model. In all cases, we find that our preferred models have lower RMSE and MAE than do the generalized linear models in Kaambwa et al. with similar independent variables. In fact, the mixture models reported here had lower RMSE than did every model reported by Kaambwa et al. that used the AQLQ-S summary score as an explanatory variable. Kaambwa et al. did not provide any visual representation of model fit for their preferred models. For this reason, we can only compare averages such as RMSE and MAE and we are unable to compare the model fit over the PBM distributions.

Mixture models are much more flexible than typically used mapping models. The methods we test constrain model outputs to the feasible range between full health and the worst health state, with the ability to have large masses at the extremes. Modeling the gap between full health and the next feasible health state adds additional flexibility and further restricts outputs to the feasible range for the utility instrument. When using these more complex models it is important to consider the characteristics of the data and to search for the most appropriate model, both through using different specifications of the models, but also ensuring that the global maximum likelihood is found [32]. In our search for a global maximum, we found other maxima that included mass points at the top of the distribution. This predicted the number of observations in full health very well but the overall fit was much worse and the graphs for the means showed clear mis-specification.

### Study Limitations

There are some limitations to our study. First, we were unable to validate our results on an external data set because no suitable data set was available. The International Society for Pharmacoeconomics and Outcomes Research good practice report on mapping does not recommend routinely splitting the sample to validate results on part of the sample [31]. Validation of results on external data sets should be encouraged when these data sets are available. Second, asthma is self-reported in the MIC data set and therefore could suffer from biased reporting. Third, the data consist of observations from across six different countries, but we use the same UK tariffs for all observations. This has been shown, however, to make little difference to results [33]. Finally, we cannot compare our results with those of Kaambwa et al. [7] beyond model fit averages such as the RMSE and the MAE because the authors do not publish any information on the model fit at different parts of the PBM distributions.

## Conclusions

Our results show that each of the chosen models is an improvement on more traditionally used linear predictions. Both types of mixture model used in this study are able to closely fit the data without the biased performance characteristic of many commonly used mapping approaches. The beta-based mixture models outperformed the ALDVMM models but at the expense of increasing the number of parameters as well as estimation time. Skilled judgment is critical in determining the optimal model. Caution is required in ensuring that a truly global maximum likelihood has been identified.

Previous cost-effectiveness analysis, carried out by the National Institute of Health and Care Excellence, has used mapping algorithms to estimate PBMs using non–preference-based disease-specific measures in patients with asthma [4], [6]. The mapping algorithms resulting from this study produce estimates that do not suffer from bias and fit the data well at all parts of the PBM distributions. The algorithms provided with this study can be used with confidence in applied cost-effectiveness analysis.

## Footnotes

Supplemental data associated with this article can be found in the online version at http://dx.doi.org/10.1016/j.jval.2017.09.017.
